# Evaluation of bone-protecting effects of palm carotene mixture in two- and three-dimensional osteoblast/osteoclast co-culture systems

**DOI:** 10.7150/ijms.103445

**Published:** 2025-01-06

**Authors:** Michelle Min-Fang Yee, Kok-Yong Chin, Soelaiman Ima-Nirwana, Ekram Alias, Kien Hui Chua, Sok Kuan Wong

**Affiliations:** 1 Department of Pharmacology, Faculty of Medicine, Universiti Kebangsaan Malaysia, Jalan Yaacob Latif, Bandar Tun Razak, 56000 Cheras, Kuala Lumpur, Malaysia.; 2 Department of Biochemistry, Faculty of Medicine, Universiti Kebangsaan Malaysia, Jalan Yaacob Latif, Bandar Tun Razak, 56000 Cheras, Kuala Lumpur, Malaysia.; 3 Department of Physiology, Faculty of Medicine, Universiti Kebangsaan Malaysia, Jalan Yaacob Latif, Bandar Tun Razak, 56000 Cheras, Kuala Lumpur, Malaysia.

**Keywords:** bone cells, bone remodeling, osteoporosis, provitamin A, scaffold

## Abstract

**Background:** Carotene exists naturally in a complex mixture consisting of alpha (α), beta (β), and gamma (γ)-isoforms. Previous studies investigated the effects of individual carotene isomers on bone rather than their actions in a mixture.

**Purpose:** This study explored the bone-protective properties of palm carotene mixture using both two- and three-dimensional co-culture systems.

**Study design:** The viability of human foetal osteoblasts (hFOB 1.19), viability of human monocytic cell line (THP-1), osteoblast differentiation, osteoclast maturation, bone quality and strength were assessed in two- and three-dimensional co-culture system after treatment of palm carotene mixture.

**Methods:** The viability of hFOB 1.19 and THP-1 was determined on day 1, 3, and 6 following treatment of palm carotene mixture. The osteoblast-osteoclast co-culture (ratio of hFOB 1.19 to THP-1 = 2:1) was treated with palm carotene mixture as well as subjected to alkaline phosphatase (ALP) and tartrate resistant acid phosphatase (TRAP) staining on day 21 to assess the osteoblast proliferation and osteoclast maturation. Dual-energy X-ray absorptiometry, micro-computed tomography, universal testing machine, and bone histomorphometry were used to assess the bone parameters of scaffolds co-cultured with osteoblasts and osteoclasts.

**Results:** Palm carotene mixture (3.13 - 50 μg/mL) increased osteoblast viability. Monocyte viability decreased in lower concentration (3.13 - 12.5 μg/mL) but increased in higher concentration (25 - 50 μg/mL) of palm carotene mixture. Treatment with palm carotene mixture (12.5 µg/mL) demonstrated earlier peak for the ALP-positive area on day 14 but decreased total number of TRAP-positive multinucleated cells on day 21. Palm carotene mixture also increased bone volume and osteoblast number in the three-dimensional co-culture system.

**Conclusion:** Palm carotene mixture potentially exhibits beneficial effects on bone by accelerating osteoblast proliferation and suppressing osteoclast maturation. The findings of current study serve as the basis for the further validation through animal experiments and human trials.

## Introduction

Bone is a natural three-dimensional mineralised structure made up of cells, vessels, collagen and mineral-forming hydroxyapatite in its extracellular matrix 1. A tightly regulated bone remodeling process entailing old or damaged bones are replaced by new bones is crucial to maintain the structural integrity of skeletal system to endure mechanical load and strain. Osteoblasts and osteoclasts are responsible for governing bone formation and resorption activities, respectively. Increased bone resorption and/or lifespan of osteoclasts as well as decreased bone formation and/or lifespan of osteoblasts result in osteoporosis. The medications for osteoporosis treatment include bisphosphonates, hormone replacement therapy, selective oestrogen receptor modulators, parathyroid hormone analogues as well as monoclonal antibody targeting receptor activator of nuclear-kappa B ligand (RANKL) and sclerostin. Osteoporosis prevention option is limited to calcium and vitamin D supplements. The use of these pharmacological therapeutic and prophylactic agents is associated with adverse events. Research is ongoing to identify and develop nutraceuticals with anabolic and/or anti-osteoporotic properties as adjunct therapy to overcome the drawbacks of current treatments and limited option of preventive agents for osteoporosis.

Vitamin A is a lipid-soluble compound that can be consumed as preformed retinol or provitamin A. Carotenes are provitamin A carotenoids that can be converted to vitamin A in the body. They are commonly found in plants such as fruits and vegetables 2. The health benefits of carotenes on bone have been studied, with attention has been paid on the effects of individual isomers mainly α-carotene or β-carotene 3. Diet containing 0.025% β-carotene increased bone mineral density (BMD) in the hindlimb unloading mice 4. *In vitro*, treatment with β-carotene enhanced osteoblast differentiation and osteogenic gene expression in mouse pre-osteoblastic cells 5. The combination of β-carotene and isoflavones has been demonstrated to enhance early osteoblastic differentiation and osteogenic expression in mouse pre-osteoblastic cells 6. Another *in vitro* study reported that β-carotene inhibited cell viability and induced cell damage of bone marrow-derived monocytes/macrophages. Upon stimulated with RANKL, the formation of mature osteoclasts and resorption pits were decreased in the presence of β-carotene 7. Several human epidemiological studies pointed out that greater β-carotene intake and serum β-carotene concentration were associated with higher whole body, total hip, lumbar spine, and femoral neck BMD 8-10 as well as lower risk of hip fracture 11. Mechanistically, β-carotene attenuated the activation of nuclear factor-kappa B (NF-κB) 7, a key transcription factor for osteoclast formation in response to RANKL and inflammatory cytokines. In addition, some studies depicted higher systemic α-carotene level was associated with better bone health. Zhang *et al.* found higher BMD at whole body and hip region in individual with higher serum α-carotene concentration 12. Results obtained from an observational study indicated that osteoporotic patients had a lower α-carotene level in plasma 13.

In nature, carotene exists in a complex mixture containing various isomers. At this juncture, the bone-protecting properties of palm carotene mixture have not been investigated. Palm oil is the richest source of natural carotene with the highest ratio of α-carotene as compared to carotene isolated from other sources 14. Carotene is more superior in its α-isomer than β-isomer for several considerations: (a) the bioavailability of α-carotene is greater than β-carotene 15; (b) α-carotene has greater antioxidant capacity than β-carotene 16; and (c) the conversion efficacy of β-carotene to vitamin A (retinol) is twice higher than α-carotene 17. High doses of retinoic acid inhibit osteoblast differentiation and mineralisation accelerating bone loss 18. Thus, the α-isomer may be superior to β-isomer in protecting the bone.

Preclinical *in vitro* model mimicking cellular and tissue functioning in humans is essential to represent the *in vivo* complexity. Jolly *et al.* optimised a reproducible two-dimensional co-culture system utilising human foetal osteoblasts (hFOB 1.19) and peripheral blood mononuclear cells at a ratio of 2:1 19. This cell configuration ensures good interaction between bone-forming osteoblasts and bone-resorbing osteoclasts to achieve balanced bone remodelling. Besides, it does not require exogenous stimuli such as macrophage colony-stimulating factor (M-CSF) and RANKL to promote osteoclast proliferation and maturation 19. Subsequently, the same group of researchers developed a three-dimensional co-culture system comprising the seeding of osteoblasts and osteoclasts on bovine bone scaffold that resembles the physiological condition of human bone. They suggested that this system is considered as a cost-effective model demonstrating structural, functional, and mechanical similarities with natural bone. It is suitable as an *in vitro* model for screening agents with bone-protecting action 20. The characteristics of an excellent bone scaffold include osteoconductive (to allows adhesion of cells to surface), osteogenic (to support new bone formation), biocompatible (to allow cell activity without adverse cell response), biodegradable (to decompose easily), interconnected porous microarchitecture (for excellent cell adhesion, migration, proliferation and differentiation), and great mechanical strength (to bear weight) 21-24. In this regard, the trabecular bone region fulfils the features of a bone scaffold possessing a high surface-to-volume ratio that facilitates dynamic microstructural alterations and bone metabolism activity.

In this study, we investigated the effects of palm carotene mixture on the cell viability of osteoblasts and monocytes. The effects of palm carotene mixture on osteoblast differentiation and osteoclast maturation were then evaluated using a two-dimensional osteoblast-osteoclast co-culture system. Subsequently, the skeletal-protecting effects of palm carotene mixture were determined using a three-dimensional co-culture system resembling the human bone microenvironment. We hypothesised that palm carotene exerts advantageous effects on improving bone health, potentially serving as a natural anti-osteoporotic agent for addressing bone-related disorders.

## 2. Materials and methods

### 2.1 Cell culture

Human foetal osteoblasts, hFOB 1.19 (Product code: CRL-11372^TM^) and human monocytic cell line, THP-1 (Product code: TIB-202^TM^) were purchased from American Type Culture Collection (ATCC, Manassas, USA). The hFOB 1.19 cells were cultured in Dulbecco's Modified Eagle Medium/Nutrient Mixture F-12 (DMEM/F-12) (Sigma Aldrich, St. Louis, USA) whereas the THP-1 cells were cultured in Roswell Park Memorial Institute (RPMI 1640) (ATCC, Manassas, USA). Both media were supplemented with 10% fetal bovine serum (Sigma Aldrich, St. Louis, USA) and 1% penicillin-streptomycin (Nacalai Tesque Inc., Kyoto, Japan). Healthy hFOB 1.19 and THP-1 cells at passage 6 and 4 respectively with 90% confluency were harvested. Cells were cultured under aseptic techniques and incubated under 5% carbon dioxide (CO_2_) and ≥ 97% humidity.

### 2.2 Treatment preparation

Palm carotene mixture (EVTene^TM^ 20%, Batch number: B1/20/0357_1_150719; molecular formula: C_40_H_56_; molecular weight: 536.88 g/mol), isolated from *Elaeis guineensis*, was a kind gift from ExcelVite Sdn. Bhd (Chemor, Malaysia). The mixture consists of 20.8% mixed carotene complex (comprising of α-carotene, β-carotene, γ-carotene, and lycopene) as well as 79.2% red palm olein (comprising of monoglyceride, diglyceride, and triglyceride). The ratio for α-carotene, β-carotene, and other carotenoids in the mixture is 33:66:1. Palm carotene mixture of various concentrations (3.13 - 50 µg/mL) was prepared by dissolving in <0.1% tetrahydrofuran (Sigma Aldrich, St. Louis, USA). FOSAMAX Plus^®^ (Merck, Rahway, USA) served as the positive control. Each tablet contains 91.37 mg of alendronate monosodium salt trihydrate and 140 μg of cholecalciferol (equivalent to 5600 IU vitamin D). It was diluted in distilled water to a concentration of 10 nM.

### 2.3 Cell viability assay

The viability of hFOB 1.19 cells and THP-1 cells after treated with palm carotene mixture were measured using 3-(4,5-dimethylthiazol-2-yl)-2,5-diphenyl-2H-tetrazolium bromide (MTT) assay. A total of 3.33 x 10^4^ cells were treated with palm carotene mixture at the concentrations of 3.13, 6.25, 12.5, 25, and 50 µg/mL. On day 1, 3 and 6, MTT solution at concentration of 0.5 mg/mL was added into the cells and incubated for 4 hours at 37℃ in humidified atmosphere with 5% CO_2_. The absorbance was measured at 570 nm using a microplate reader (Tecan, Mannedorf, Switzerland) after dimethyl sulfoxide (Sigma, USA) was added to the wells. The mean absorbance was used to calculate the percentage of cell viability as the following: percentage of cell viability = (A_treatment_ - A_blank_)/(A_control_-A_blank_) x 100%, where 'A' refers to the absorbance.

### 2.4 Alkaline phosphatase (ALP) and tartrate-resistant acid phosphatase (TRAP) staining in two-dimensional osteoblast-osteoclast co-culture system

The hFOB 1.19 (9.99 x 10^4^) and THP-1 (4.99 x 10^4^) cells were cultured in a ratio of 2:1 respectively in DMEM/F-12 (Sigma Aldrich, St. Louis, USA). The co-cultured hFOB 1.19 and THP-1 cells were stained using ALP and TRAP staining kits (Solarbio® Life Science, China) on day 7, 14, and 21 as per manufacturers' instructions. Images of cells were taken at 100x magnification with EVOS^TM^ Cell Imaging System (Invitrogen, California, USA). The ALP-positive site was indicated by blue stain located in the cytoplasm whereas the TRAP-positive multinucleated cells with more than three nuclei were counted as mature osteoclasts.

### 2.5 Preparation of bovine bone scaffolds

Bovine femurs were procured from local slaughterhouse. Trabecular region at the metaphysis of bovine femurs was sectioned into blocks with dimension of 5 mm x 5 mm x 5 mm using a bone saw machine (PrimeHub W210CA Stainless Steel Meat Bone Saw Machine, China). Bone scaffolds were standardised for their porosity (70 - 90%) using micro-computed tomography (micro-CT). Bone scaffolds were randomised into five experimental groups: (a) native bone scaffolds (NB), (b) osteoporotic bone scaffolds (OB), (c) negative control bone scaffolds (NC), (d) 12.5 μg/mL palm carotene mixture-treated bone scaffolds (TB), and (e) 10 nM alendronate-treated bone scaffolds (positive control, PC). The NB group was native bovine bone scaffolds without undergoing decellularisation and demineralisation. The bone scaffolds of OB, NC, TB, and PC groups were subjected to decellularisation using four freeze-thaw cycles (consisting of freezing at -80℃ for 1 day and thawing at 121℃ for 20 minutes in sequence) followed by non-ionic detergent treatment using Triton X-100 (Sigma, USA). Subsequently, the bone scaffolds were demineralised using 0.6 N hydrochloric acid (R&M Chemicals, Malaysia) for 1 hour at room temperature. For NC, TB, and PC groups, the bone scaffolds were reseeded with osteoblast/osteoclast co-culture. The absence of bovine bone cells (osteoblasts, osteoclasts and osteocyte) in the OB group as compared to the NB group ensures successful decellularisation and demineralisation. All scaffolds were sterilised before use.

### 2.6 Plasma collection

Blood was collected via venipuncture from 10 male volunteers aged 23 - 30 years old without any underlying medical conditions using anticoagulant ethylenediaminetetraacetic acid (EDTA) tubes. Plasma was collected via centrifugation at 3500 rpm for 10 minutes. Plasma was used for fibrin formation to entrap osteoblasts onto the bone scaffold surface in the three-dimensional system. The protocol was reviewed and approved by Research Ethics Committee (REC) Universiti Kebangsaan Malaysia (ethical approval code: UKM PPI/111/8/JEP-2020-603).

### 2.7 Osteoblast-osteoclast co-culture in bovine bone scaffolds

A total of 2 x 10^6^ hFOB 1.19 cells, 2 mL plasma, and 0.5 M calcium chloride (Sigma, USA) were mixed and seeded on each bovine bone scaffold. Agarose gel (Sigma, USA) was prepared to fix the bone scaffold in place. Monocytes (1 x 10^6^ cells) were injected into bone scaffold on the third day. Co-cultured cells were incubated in DMEM/F-12 complete medium (Sigma Aldrich, St. Louis, USA) and treatment was given under standard culture conditions (37℃, 5% CO_2_ and ≥97% humidity). The culture medium was replaced every three days. Bone scaffold was harvested on day 21 for subsequent analyses.

### 2.8 Dual energy X-ray absorptiometry (DXA) analysis of bovine bone scaffolds

The BMD and bone mineral content (BMC) of the bovine bone scaffolds were measured using a DXA machine (QDR-4500, Hologic Inc, Bedford, USA) and analysed using QDR system software (Hologic Inc, Bedford, USA).

### 2.9 Micro-CT analysis of bovine bone scaffolds

Bone scaffolds were scanned using micro-CT machine (Skyscan 1172, Bruker, Belgium). Acquisition settings for the scanning were standardised as follows: X-ray voltage (92 kV), X-ray current (100 µA), filter (0.5 mm aluminium), rotation step (0.5°), image pixel size (13.9 µm), camera resolution (high), frame averaging (2) and integration time (3000 ms). Image reconstruction was done after scanning using NRecon software (V1.6.10.4, Skyscan, Bruker, Belgium), followed by image reorientation using Data Viewer software (V1.5.2.4, Skyscan, Bruker, Belgium). Semi-automated contouring was done on 200 slices volume of interest (consisting of 100 slices above and 100 slices below the midpoint of scaffold images) using CTAn (V1.16.1.0+, Skyscan, Bruker, Belgium). Parameters obtained from micro-CT analysis were bone volume/tissue volume (BV/TV, unit: %), trabecular number (Tb.N, unit: mm^-1^), trabecular thickness (Tb.Th, unit: mm), trabecular separation (Tb.Sp, unit: mm), and porosity (unit: %). Three-dimensional bone images were generated using CTVox software (V1.16.1.0+, Skyscan, Bruker, Belgium).

### 2.10 Compression test of bovine bone scaffolds

Compression test was done using Shimadzu universal testing machine and were analysed using Trapezium Lite X software (Autograph AGS-X 500N Shimadzu, Kyoto, Japan). During the test, compressive load at a velocity of 10 mm/min were applied on the scaffold until fracture occurred. Parameters obtained from compression test were load (unit: N), displacement (unit: mm), stiffness (unit: N/mm), stress (unit: N/mm^2^), strain (unit: %) and Young's modulus (unit: N/mm^2^) of the bone scaffolds.

### 2.11 Histomorphometry analysis of bovine bone scaffolds

For the evaluation of static bone histomorphometry, the harvested bone scaffolds were fixed in phosphate buffered formalin followed by EDTA for decalcification (2 weeks). Bone scaffolds were rinsed with water as well as treated with ascending concentrations of ethanol (Hamburg, Germany), mixed ethanol and toluene (1:1) (R&M Chemicals, Malaysia) and absolute toluene. Next, bone scaffolds were immersed in paraffin wax (Leica Biosystems, Jerman) for 3 times (3 hours each time), embedded and frozen at -20℃ before slicing them into sections. The bone sections were stained using haematoxylin and eosin (H&E) staining kit (Leica Biosystems, Germany) as per manufacturers' instructions. Images of decalcified sections were taken at 400x magnification using a light microscope (Olympus, Japan) equipped with Image-PRO Plus software version 5.0.2.9. Parameters obtained from H&E staining were osteoblast number/bone area (Ob.N/BA, unit: number per bone area in mm^2^), osteoclast number/bone area (Oc.N/BA, unit: number per bone area in mm^2^), eroded surface/bone surface (ES/BS, unit: %), osteoid surface/bone surface (OS/BS, unit: %), and osteoid volume/bone volume (OV/BV, unit: %).

For the evaluation of structural bone histomorphometry, bone scaffolds were immersed in phosphate buffered formalin (Fischer Scientific, USA) for 48 hours. Bone scaffolds underwent dehydration and treated with increasing concentrations of ethanol (70%, 90%, and 100%) (Hamburg, Germany). Bone scaffolds were then cleared using xylene (Sigma, USA), embedded in methyl methacrylate using glass bottles, sectioned using microtome, and stained using von Kossa method. Briefly, bone sections were treated with acetone (HmBG Chemicals, Germany), descending concentrations of ethanol (100%, 96%, and 70%), and rinsed with water. Sections were treated with 1% silver nitrate (Sigma, USA), exposed to light for 20 minutes, treated with 2.5% sodium thiosulphate (Sigma, USA), as well as ascending concentrations of ethanol (70%, 96%, and 100%). Slides were cleared using diethyl ether (HmBG Chemicals, Germany), dropped with dibutylphthalate polystyrene xylene (Sigma, USA), and covered using cover slip for observation under a light microscope (Olympus, Japan) equipped with Image-PRO Plus software version 5.0.2.9. Parameters obtained from von Kossa staining were BV/TV (unit: %), Tb.Th (unit: mm), Tb.N (unit: mm^-1^), and Tb.Sp (unit: mm).

### 2.12 Statistical analysis

All *in vitro* experiments were performed in triplicates, a common practice of scientific experiments to improve the precision of parameter estimates. The statistical analyses were performed using Statistical Package for the Social Sciences (SPSS) version 26 (IBM Corporation, Armonk, NY, USA). All values were presented as mean ± standard error of mean (SEM). The normality of the data was assessed using Shapiro-Wilk test. Normally distributed data with single time-point were analysed using one-way analysis of variance (ANOVA) with Tukey's or Dunnett's post-hoc pairwise comparison test depending on the homogeneity of variance. Mixed design ANOVA with small effect analysis as post hoc pairwise comparison test was used for normally distributed data with multiple time-points. A p-value of <0.05 was considered statistically significant.

## 3. Results

### 3.1 Effects of palm carotene mixture on the viability of hFOB 1.19 cells and THP-1 monocytes

**Figure 1:**
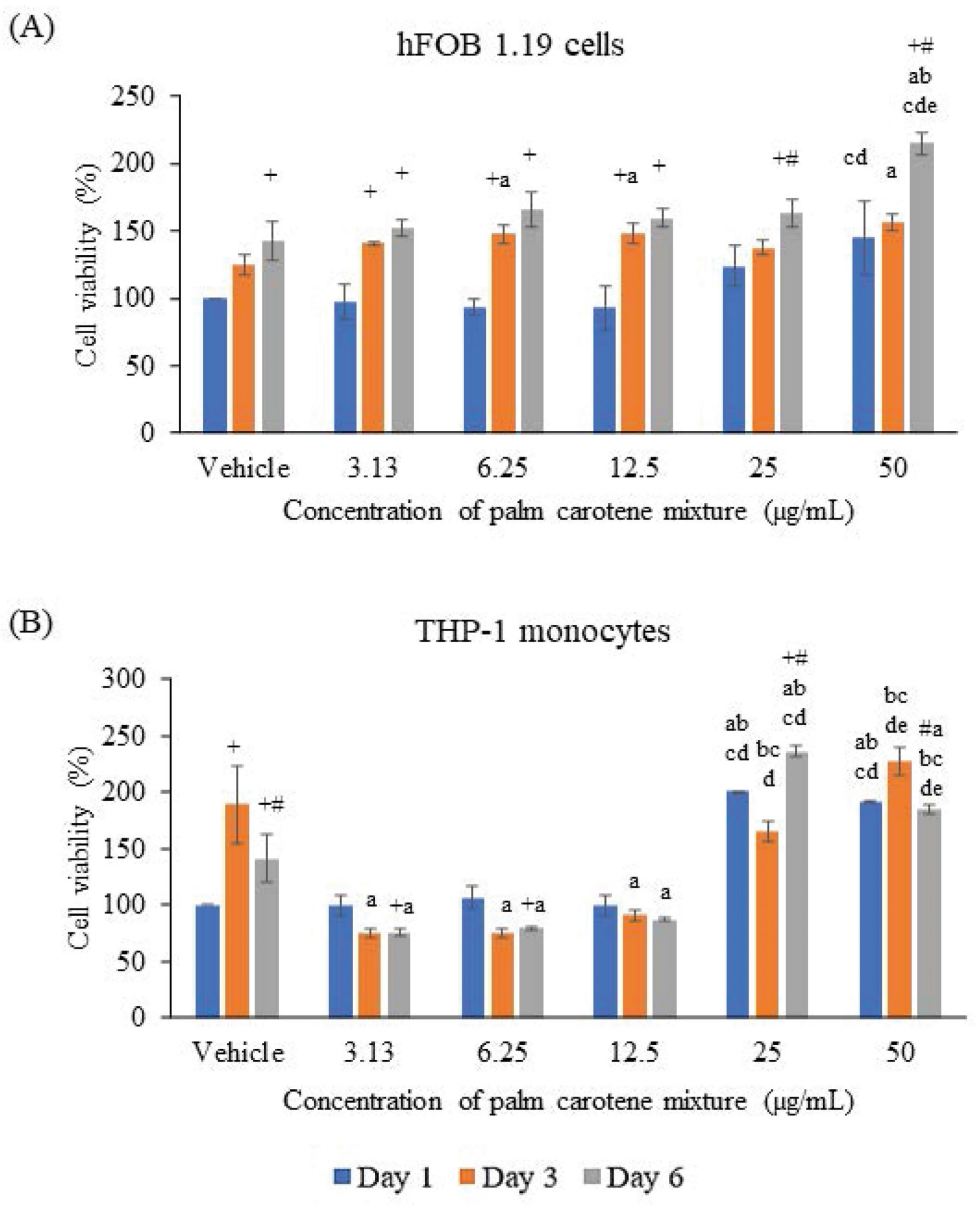
The viability of (A) hFOB 1.19 and (B) THP-1 cells treated with palm carotene mixture at different concentrations (0 - 50 µg/mL). Data are expressed as mean ± SEM. '+' indicates significant difference with day 1; '#' indicates significant difference with day 3; 'a' indicates significant difference with vehicle; 'b' indicates significant difference with 3.13 µg/mL palm carotene mixture; 'c' indicates significant difference with 6.25 µg/mL palm carotene mixture; 'd' indicates significant difference with 12.5 µg/mL palm carotene mixture; 'e' indicates significant difference with 25 µg/mL palm carotene mixture.

The hFOB 1.19 cells were seen viable at all treatment concentrations of palm carotene mixture ranging from 3.13 - 50 µg/mL. For within-group comparison, the non-treated cells showed significant increase on day 6 as compared to day 1 (p<0.05). Treatment with 3.13, 6.25, and 12.5 µg/mL palm carotene mixture significantly increased cell viability on day 3 and day 6 relative to day 1 (p<0.05). For cells treated with 25 and 50 µg/mL palm carotene mixture, cells were observed to increase on day 6 in comparison to day 1 and 3 (p<0.05). For between-group comparison, the cells treated with 50 µg/mL of palm carotene mixture showed significant higher cell viability as compared to those treated with 6.25 and 12.5 µg/mL of palm carotene mixture on day 1 (p<0.05). On day 3, the groups treated with 6.25, 12.5, and 50 µg/mL of palm carotene mixture had significant higher cell viability as compared to the control group (p<0.05). On day 6, the cells treated with 50 µg/mL of palm carotene mixture showed significant increase in cell viability as compared to the non-treated cells and palm carotene mixture-treated cells at the concentrations of 3.13 - 25 µg/mL (p<0.05) (Fig. 1A).

The viability of THP-1 cells was decreased after treated with 3.13 - 12.5 µg/mL palm carotene mixture but increased in the 25 - 50 µg/mL treatment groups. The within-group analysis showed that the vehicle-treated cells viability increased from day 1 to day 3 and decreased on day 6 (p<0.05). Higher cell viability was also observed in cells treated with 25 µg/mL palm carotene mixture on day 6 than day 1 and day 3 (p<0.05). However, the cells treated with 50 µg/mL palm carotene mixture showed a significant decrease in cell viability on day 6 as compared to day 3 (p<0.05). The between-group analysis showed that the cells treated with 25 and 50 µg/mL of palm carotene mixture had higher viability as compared to the cells treated with vehicle, 3.13, 6.25, and 12.5 µg/mL palm carotene mixture on day 1 (p<0.05). On day 3 and day 6, treatment with 3.13 - 12.5 µg/mL palm carotene mixture significantly decreased cell viability in comparison to the cells treated with vehicle (p<0.05). On day 3, the group treated with 25 µg/mL palm carotene mixture exhibited higher cell viability in relative to the group treated with 3.13 - 12.5 µg/mL palm carotene mixture (p<0.05) while the group treated with 50 µg/mL palm carotene mixture had the highest cell viability among all the experimental groups (p<0.05). On day 6, the cells treated with 50 µg/mL palm carotene mixture demonstrated higher viability than those treated with vehicle, 3.13, 6.25, and 12.5 µg/mL palm carotene mixture whereas the cells treated with 25 µg/mL palm carotene mixture had the highest cell viability among all the experimental groups (p<0.05) (Fig. 1B).

### 3.2 Effects of palm carotene mixture on ALP and TRAP staining in a two-dimensional osteoblast-osteoclast co-culture system

The osteoblast/osteoclast co-culture was treated with 0 - 12.5 µg/mL palm carotene mixture. ALP and TRAP staining were performed on day 7, 14, and 21.

**Figure 2:**
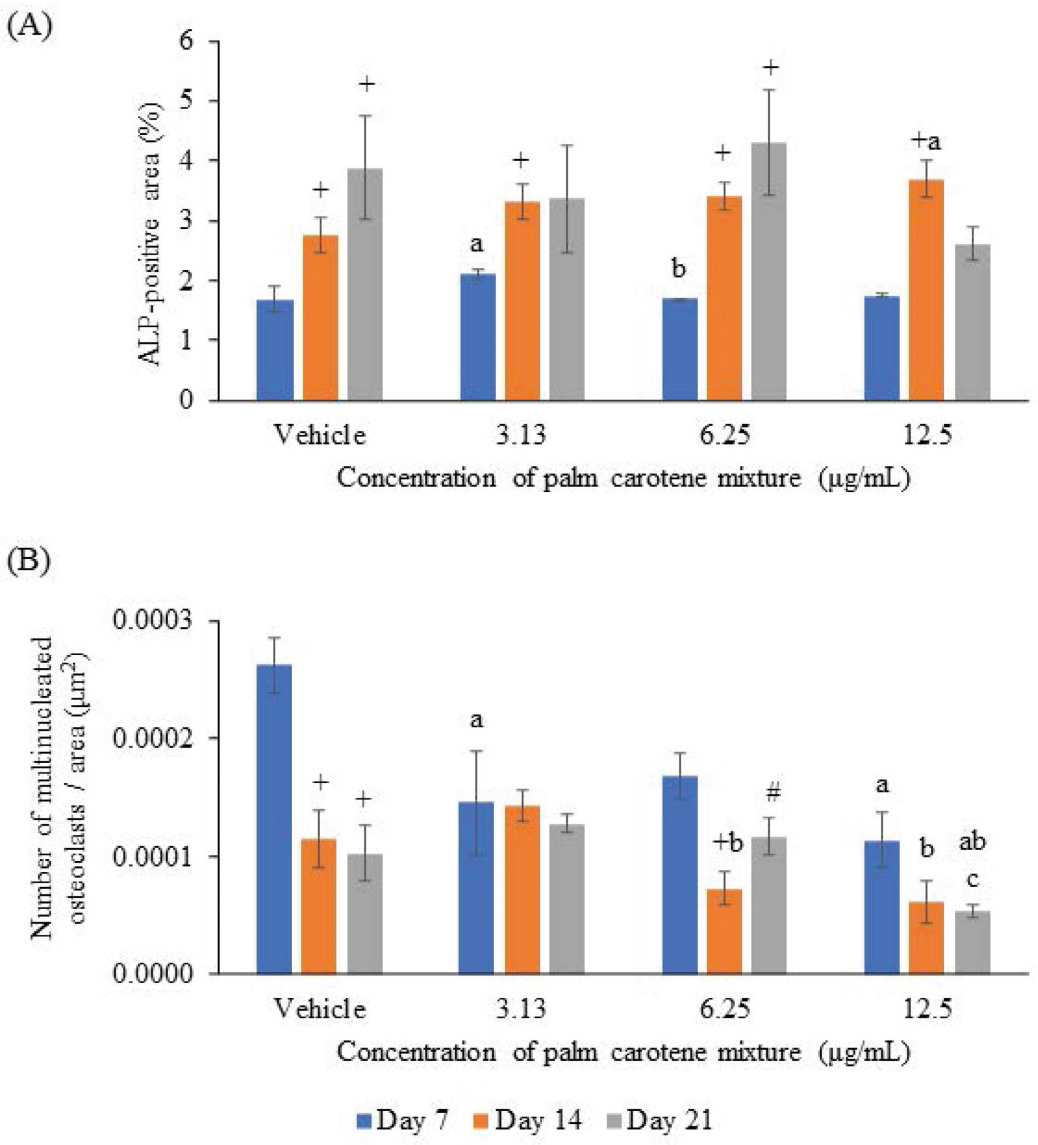
(A) ALP-positive area and (B) number of multinucleated osteoclasts in an area (μm^2^) in osteoblast/osteoclast co-culture treated with palm carotene mixture at different concentrations (0 - 12.5 μg/mL). Data are expressed as mean ± SEM. '+' indicates significant difference with day 7; '#' indicates significant difference with day 14; 'a' indicates significant difference with control; 'b' indicates significant difference with 3.13 µg/mL palm carotene mixture.

**Figure 3:**
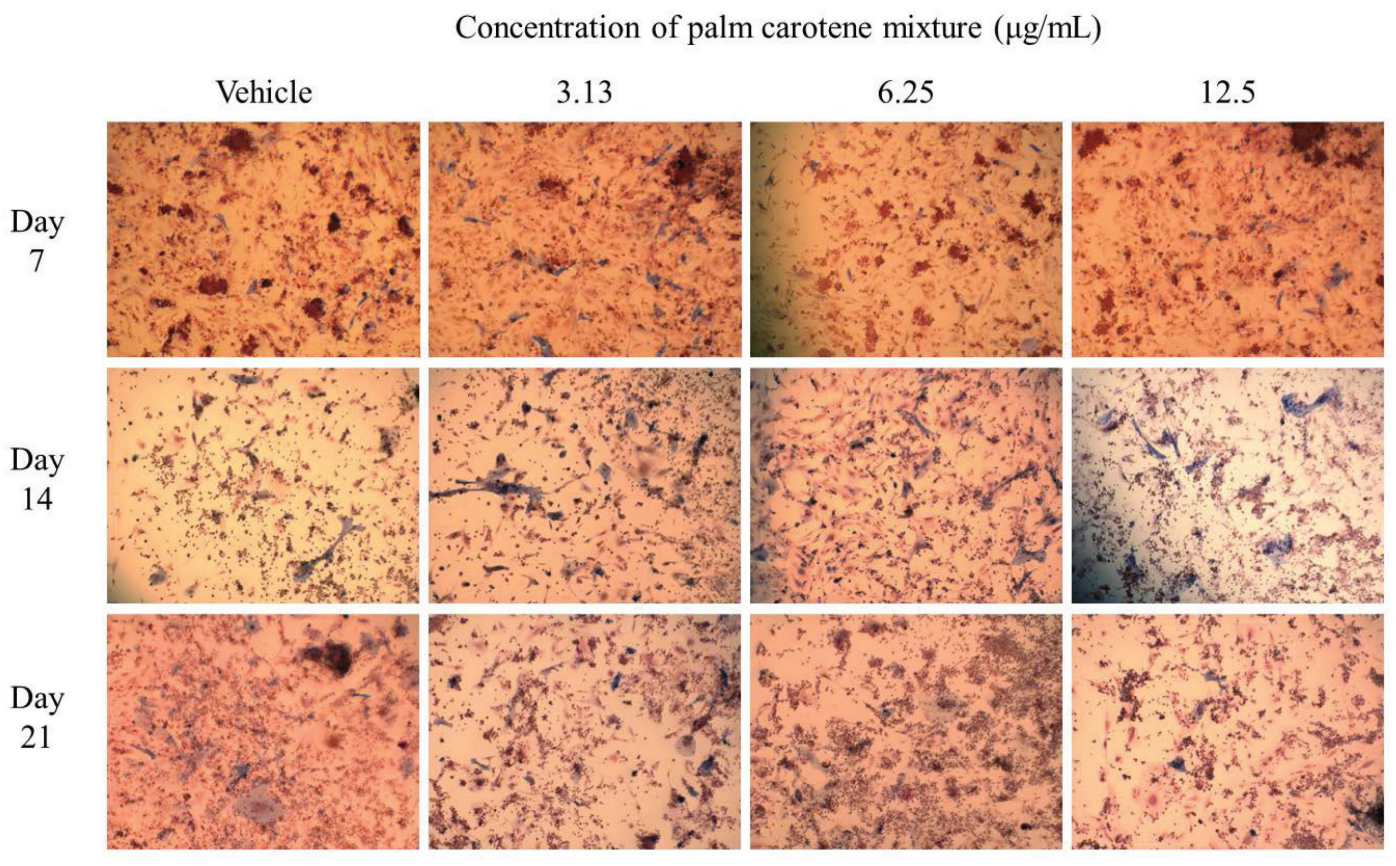
Representative images of ALP staining in osteoblast/osteoclast co-culture treated with palm carotene mixture at different concentrations (0 - 12.5 μg/mL). Microphotographs were taken at 100x magnification. Blue stain indicates ALP-positive site.

For ALP staining, the within-group analysis showed that osteoblast/osteoclast co-culture treated with vehicle and 6.25 µg/mL palm carotene mixture showed significant increases in ALP-positive area on day 14 and day 21 as compared day 7 (p<0.05). The group treated with 3.13 and 12.5 µg/mL palm carotene mixture showed significant higher ALP-positive area on day 14 as compared to day 7 (p<0.05). In between-group analysis, the co-cultured cells treated with 3.13 µg/mL palm carotene mixture showed a significant increase in ALP-positive area in comparison to those treated with vehicle on day 7 (p<0.05). The group treated with 6.25 µg/mL palm carotene mixture showed significant lower ALP-positive area than the group treated with 3.13 µg/mL palm carotene mixture on day 7 (p<0.05). On day 14, treatment with 12.5 µg/mL palm carotene mixture exhibited significant higher ALP-positive area in the co-culture as compared to the non-treated control (p<0.05). No significant difference was noted in ALP-positive area among all the experimental groups on day 21 (p>0.05) (Fig. 2A). The representative images of ALP staining in osteoblast/osteoclast co-culture without and with palm carotene mixture (3.13 - 12.5 µg/mL) treatment were depicted (Fig. 3).

**Figure 4:**
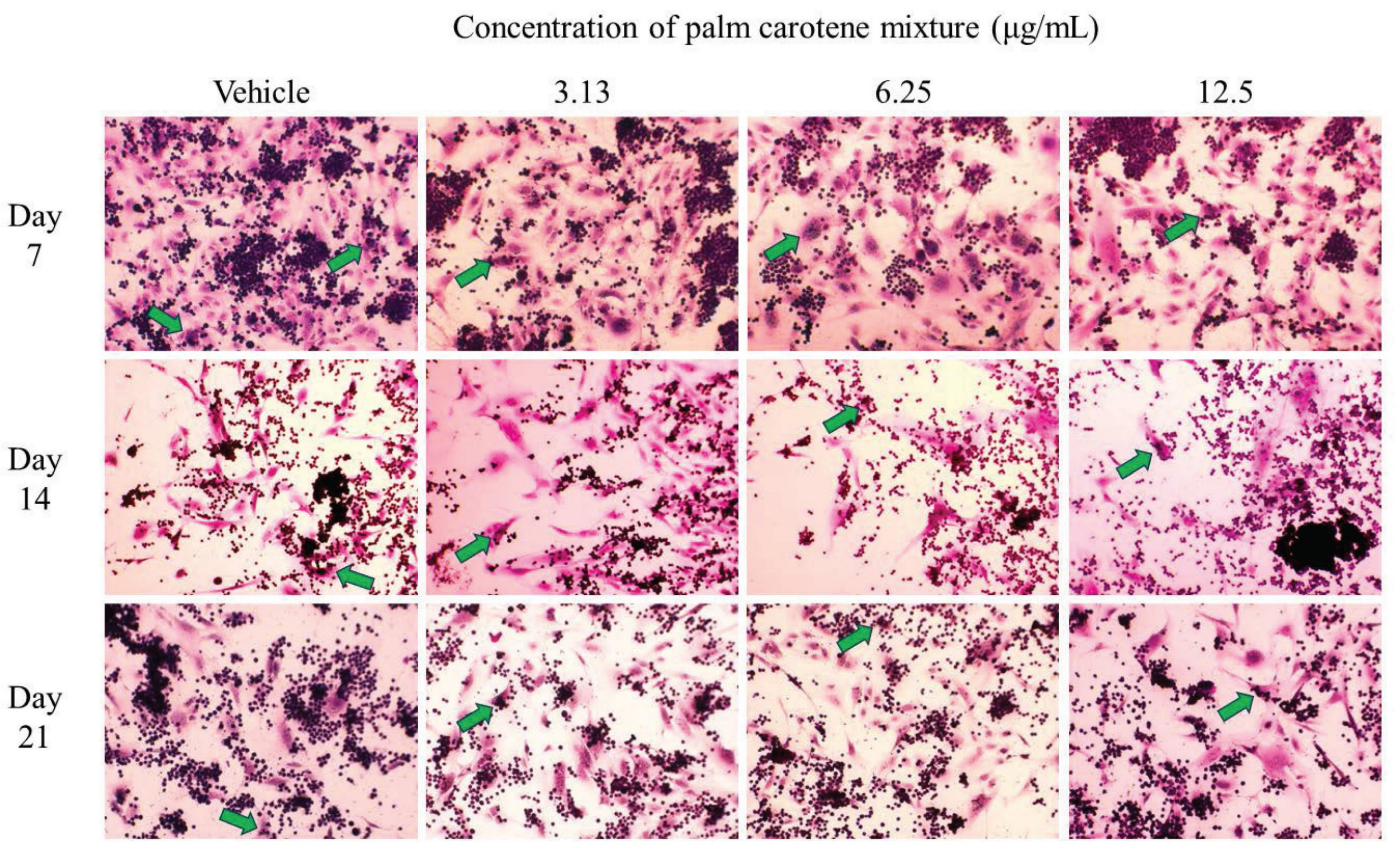
Representative images of TRAP staining in osteoblast/osteoclast co-culture treated with palm carotene mixture at different concentrations (0 - 50 μg/mL). Microphotographs were taken at 100x magnification. Green arrow indicates multinucleated osteoclasts.

For TRAP staining, the within-group analysis showed that the vehicle-treated co-culture cells had significantly lower osteoclast number on day 14 and 21 compared to day 7 (p<0.05). The co-culture cells treated with 3.13 and 12.5 µg/mL palm carotene mixture showed no changes in total number of TRAP-positive multinucleated cells from day 7 to 21 (p>0.05). There was fewer number of TRAP-positive multinucleated cells in the co-culture cells treated with 6.25 µg/mL palm carotene mixture on day 14 when compared to day 7, however there were more on day 21 when compared to day 14 (p<0.05). The between-treatment group analysis on day 7 found that the co-culture cells treated with 3.13 and 12.5 µg/mL palm carotene mixture showed significant decrease in multinucleated osteoclasts number when compared to the vehicle-treated group (p<0.05). The co-culture cells treated with 6.25 and 12.5 µg/mL palm carotene mixture showed significant decrease in the number of TRAP-positive cells compared to the co-culture cells treated with 3.13 µg/mL palm carotene mixture on day 14 (p<0.05). On day 21, the co-culture cells treated with 12.5 µg/mL palm carotene mixture showed significant reduction in osteoclasts number as compared to the co-culture cells treated with vehicle, 3.13, and 6.25 µg/mL palm carotene mixture (p<0.05) (Fig. 2B). The representative images of TRAP staining in osteoblast/osteoclast co-culture without and with palm carotene mixture (3.13 - 12.5 µg/mL) treatment were presented (Fig. 4).

### 3.3 Effects of palm carotene mixture on BMC and BMD in a three-dimensional osteoblast-osteoclast co-culture system

**Figure 5:**
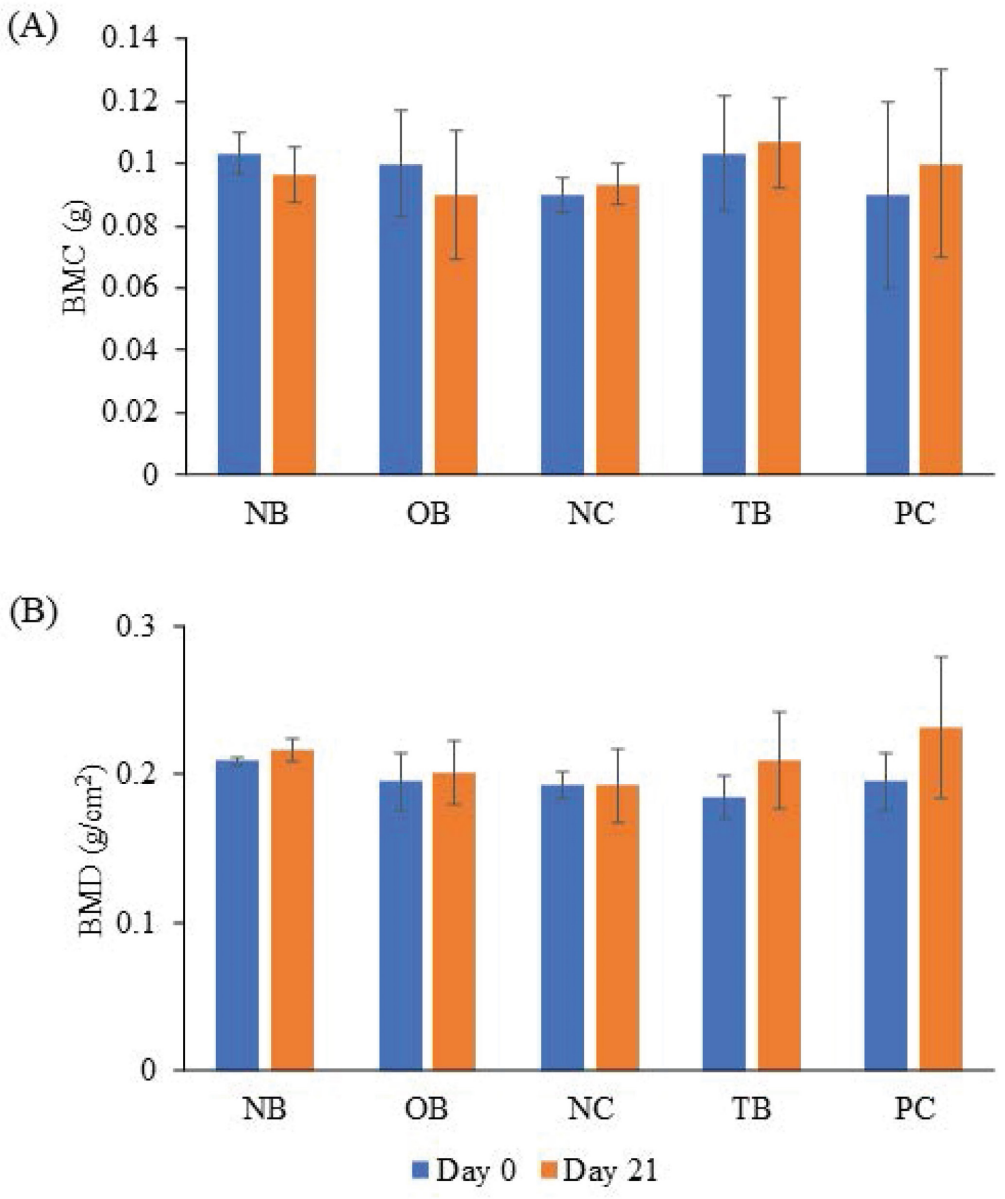
Effects of palm carotene mixture (12.5 µg/mL) on (A) BMC and (B) BMD of bone scaffold co-cultured with osteoblasts and osteoclasts for 21 days. Data are expressed as mean ± SEM. Abbreviations: NB, native bone scaffold; OB, osteoporosis bone scaffold; NC, negative control bone scaffold; TB: 12.5 μg/mL palm carotene mixture-treated bone scaffold; PC, 10 nM alendronate-treated bone scaffold.

The effects of palm carotene mixture (12.5 µg/mL) on BMC and BMD of bone scaffold co-cultured with osteoblasts and osteoclasts were evaluated after 21 days. The BMC and BMD of bone scaffolds showed no significant difference before and after treatment with palm carotene mixture among all experimental groups (p>0.05) (Fig. 5).

### 3.4 Effects of palm carotene mixture on bone microstructure in a three-dimensional osteoblast-osteoclast co-culture system

**Figure 6:**
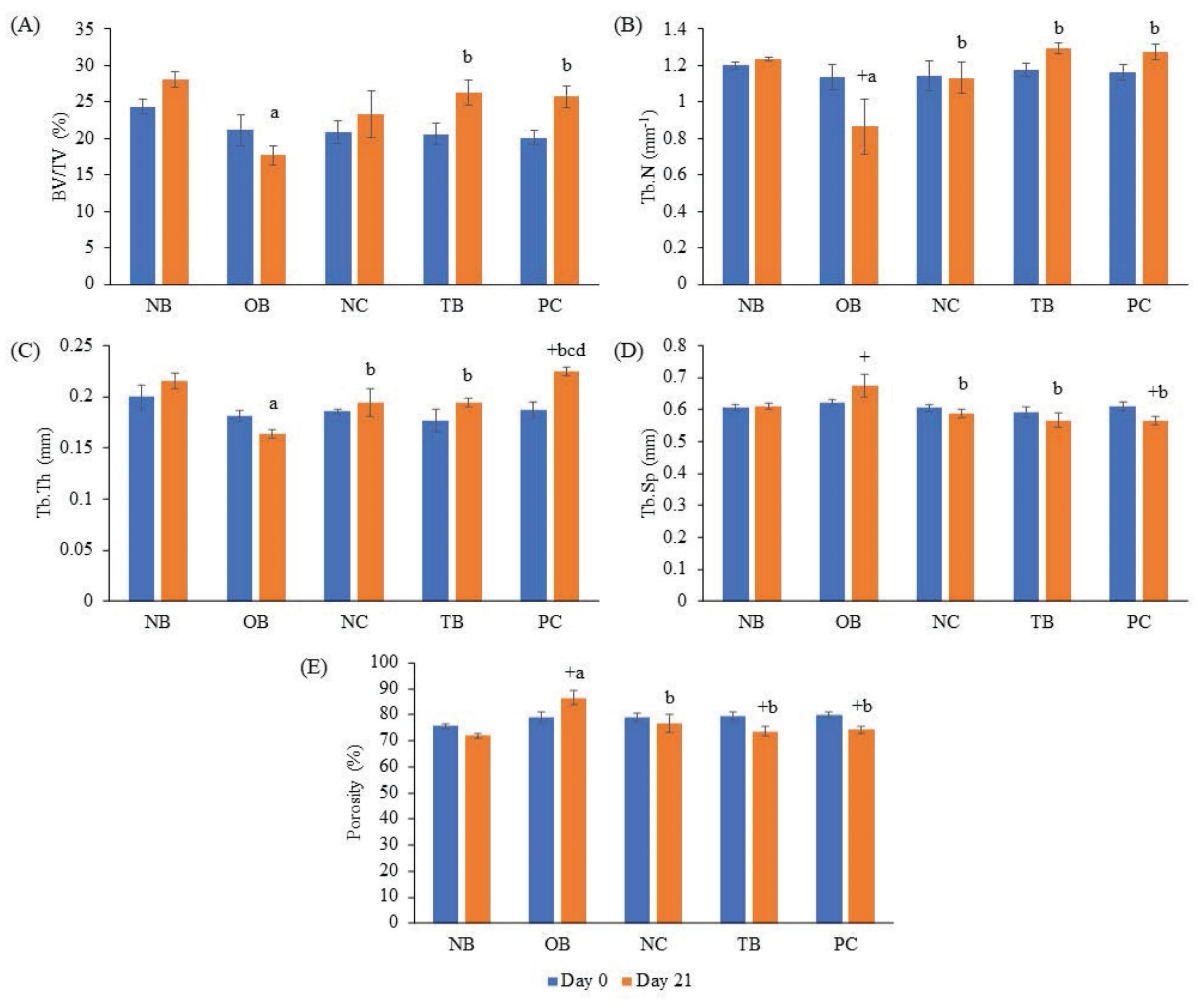
Effects of palm carotene mixture (12.5 µg/mL) on (A) BV/TV, (B) Tb.N, (C) Tb.Th, (D) Tb.Sp, and (E) porosity of bone scaffold co-cultured with osteoblasts and osteoclasts for 21 days using micro-CT. Data are expressed as mean ± SEM. '+' indicates significant difference with day 0; 'a' indicates significant difference with NB; 'b' indicates significant difference with OB. Abbreviations: NB, native bone scaffold; OB, osteoporosis bone scaffold; NC, negative control bone scaffold; TB: 12.5 μg/mL palm carotene mixture-treated bone scaffold; PC, 10 nM alendronate-treated bone scaffold.

**Figure 7:**
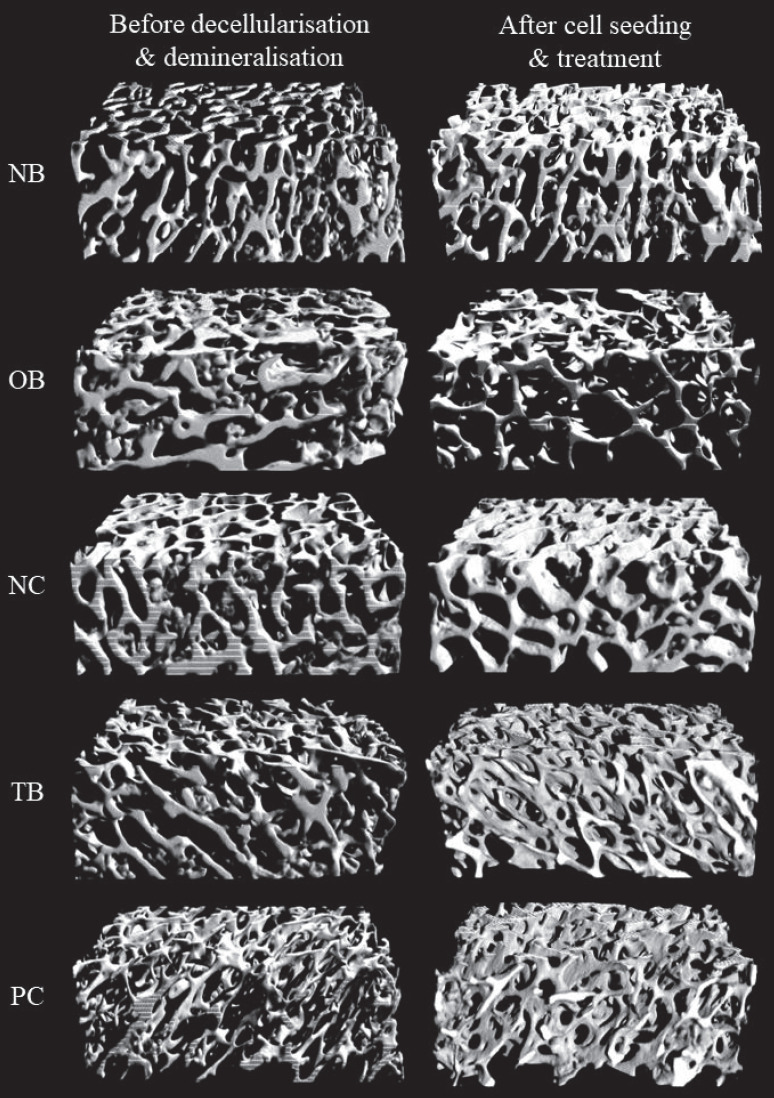
Representative of three-dimensional images via micro-CT before decellularisation and demineralisation process as well as after treatment of 12.5 µg/mL palm carotene mixture or 10 nM alendronate for 21 days. Abbreviations: NB, native bone scaffold; OB, osteoporosis bone scaffold; NC, negative control bone scaffold; TB: 12.5 μg/mL palm carotene mixture-treated bone scaffold; PC, 10 nM alendronate-treated bone scaffold.

The effects of palm carotene mixture (12.5 µg/mL) on BV/TV, porosity, Tb.N, Tb.Sp, and Tb.Th of bone scaffold seeded with osteoblasts and osteoclasts were assessed. No significant difference was observed in all bone microstructure parameters among all experimental groups before the process of decellularisation and demineralisation (p>0.05). The osteoporotic bone scaffolds (OB) had lower BV/TV, Tb.N, and Tb.Th but higher Tb.Sp and porosity when compared to the native bone scaffolds (NB) (p<0.05). The negative control bone scaffolds (NC) showed increased Tb.N and Tb.Th but decreased Tb.Sp and porosity compared to the osteoporotic bone scaffolds (OB) (p<0.05). Palm carotene mixture-treated bone scaffolds (TB) showed no changes for all micro-CT parameters as compared to the negative control bone scaffolds (NC) (p<0.05). Alendronate-treated bone scaffolds (PC) showed a significant increase in Tb.Th when compared to the negative control bone scaffolds (NC) and palm carotene mixture-treated bone scaffolds (TB) (p<0.05) (Fig. 6). Three-dimensional representative illustrations of bone scaffolds obtained from micro-CT scanning are presented (Fig. 7). Before the decellularisation and demineralisation process, the trabecular bone microstructure of bone scaffolds was similar across the experimental groups. At the end of 21-day treatment, the bone microstructure of decellularised and demineralised bone scaffolds was less dense as compared to the other groups.

### 3.5 Effects of palm carotene mixture on bone biomechanical strength in a three-dimensional osteoblast-osteoclast co-culture system

**Figure 8:**
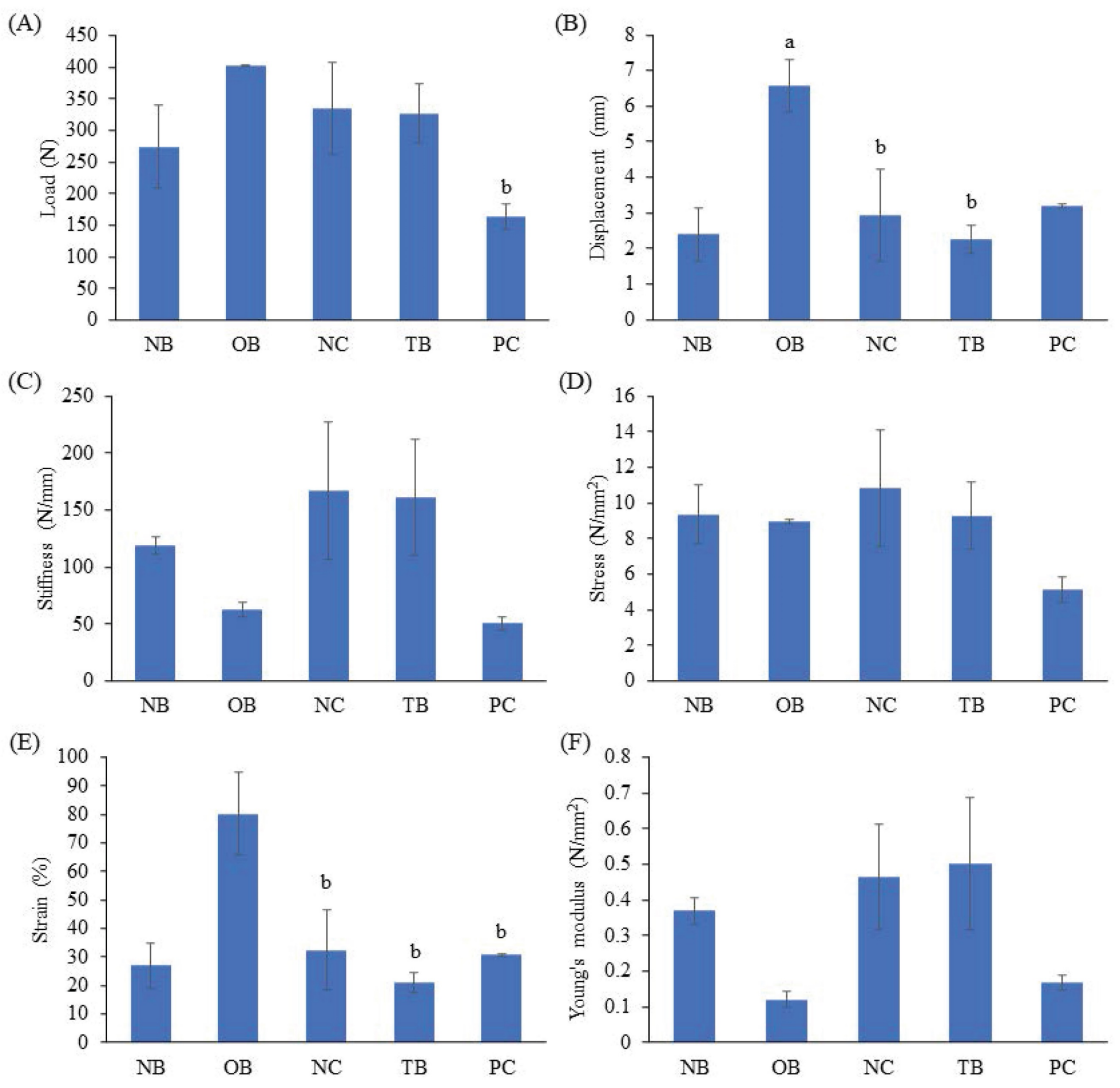
Effects of palm carotene mixture (12.5 µg/mL) on (A) load, (B) displacement, (C) stiffness, (D) stress, (E) strain, and (F) Young's modulus of bone scaffold co-cultured with osteoblasts and osteoclasts for 21 days using biomechanical strength test. Data are expressed as mean ± SEM. 'a' indicates significant difference with NB and 'b' indicates significant difference with OB. Abbreviations: NB, native bone scaffold; OB, osteoporosis bone scaffold; NC, negative control bone scaffold; TB: 12.5 μg/mL palm carotene mixture-treated bone scaffold; PC, 10 nM alendronate-treated bone scaffold.

The effects of palm carotene mixture (12.5 µg/mL) on load, displacement, stiffness, stress, strain and Young's modulus of bone scaffold seeded with osteoblasts and osteoclasts were evaluated. The osteoporotic bone scaffolds (OB) showed increased displacement and strain as compared to the native bone scaffolds (NB) (p<0.05). The negative control bone scaffolds (NC) showed decreased displacement and strain as compared to the osteoporotic bone scaffolds (OB) (p<0.05). No observable effect was observed in all biomechanical strength parameters of osteoblast/osteoclast-seeded bone scaffolds after treated with palm carotene mixture (TB) as compared to the negative control bone scaffolds (NC) (p>0.05) (Fig. 8).

### 3.6 Effects of palm carotene mixture on structural and static bone histomorphometry in a three-dimensional osteoblast-osteoclast co-culture system

**Figure 9:**
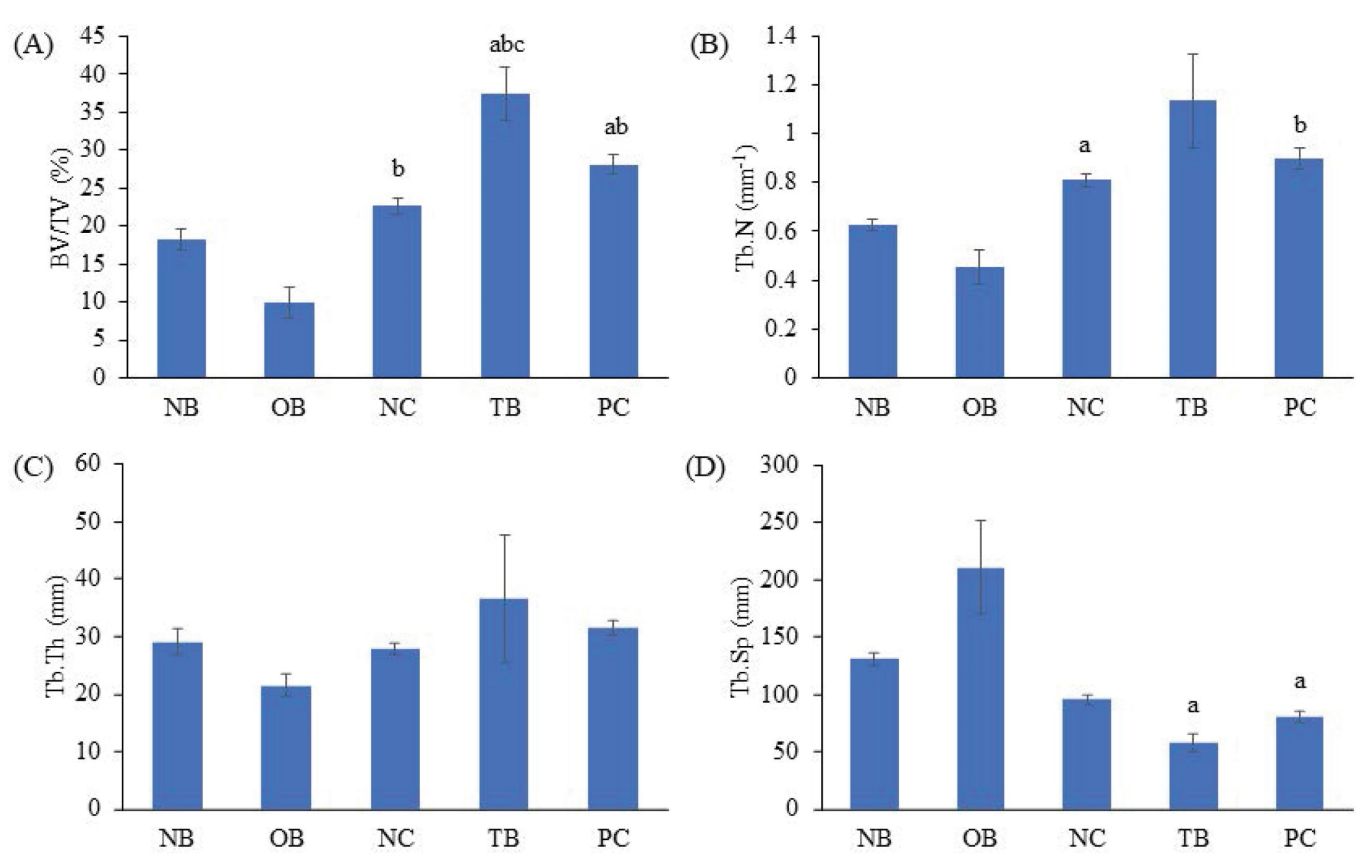
Effects of palm carotene mixture (12.5 µg/mL) on (A) BV/TV, (B) Tb.N, (C) Tb.Sp, and (D) Tb.Th of bone scaffold co-cultured with osteoblasts and osteoclasts for 21 days after von Kossa staining. Data are expressed as mean ± SEM. 'a' indicates significant difference with NB; 'b' indicates significant difference with OB; 'c' indicates significant difference with NC. Abbreviations: NB, native bone scaffold; OB, osteoporosis bone scaffold; NC, negative control bone scaffold; TB: 12.5 μg/mL palm carotene mixture-treated bone scaffold; PC, 10 nM alendronate-treated bone scaffold.

**Figure 10:**
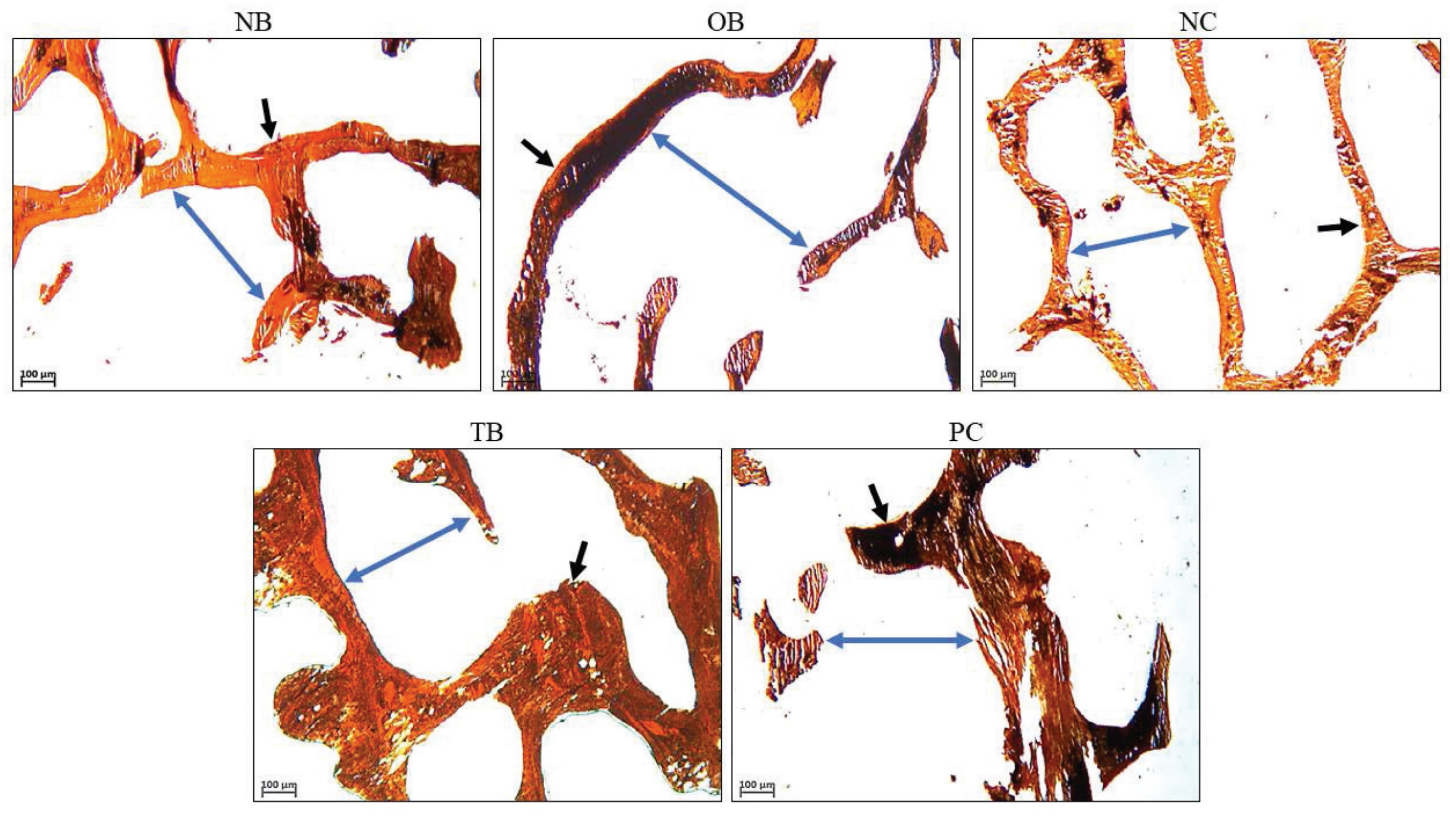
Representative images of bone scaffolds after von Kossa staining before decellularisation and demineralisation process as well as after treatment of 12.5 µg/mL palm carotene mixture or 10 nM alendronate for 21 days. The black arrows indicate trabecular bone and blue arrows indicate intertrabecular space. Abbreviations: NB, native bone scaffold; OB, osteoporosis bone scaffold; NC, negative control bone scaffold; TB: 12.5 μg/mL palm carotene mixture-treated bone scaffold; PC, 10 nM alendronate-treated bone scaffold.

The effects of palm carotene mixture (12.5 µg/mL) on BV/TV, Tb.N, Tb.Th, and Tb.Sp of bone scaffolds seeded with osteoblasts and osteoclasts after von Kossa staining were assessed. The osteoporotic bone scaffolds (OB) showed no significant difference in all parameters as compared to the native bone scaffolds (NB) (p>0.05). The negative control bone scaffolds (NC) showed higher BV/TV as compared to the osteoporotic bone scaffolds (OB) (p<0.05). Treatment of palm carotene mixture (TB) showed an increase in BV/TV when compared to the negative control bone scaffolds (NC). However, treatment with alendronate (PC) did not exert any effect on all parameters as compared to the negative control bone scaffolds (NC) (p>0.05) (Fig. 9). The representative of von Kossa staining images observed under microscope for all groups were presented (Fig. 10). The trabecular bone was thicker and the intertrabecular space was smaller in the bone scaffolds treated with palm carotene mixture (TB) or alendronate (PC) as compared to the native (NB), osteoporotic (OB), and negative control bone scaffolds (NC).

**Figure 11:**
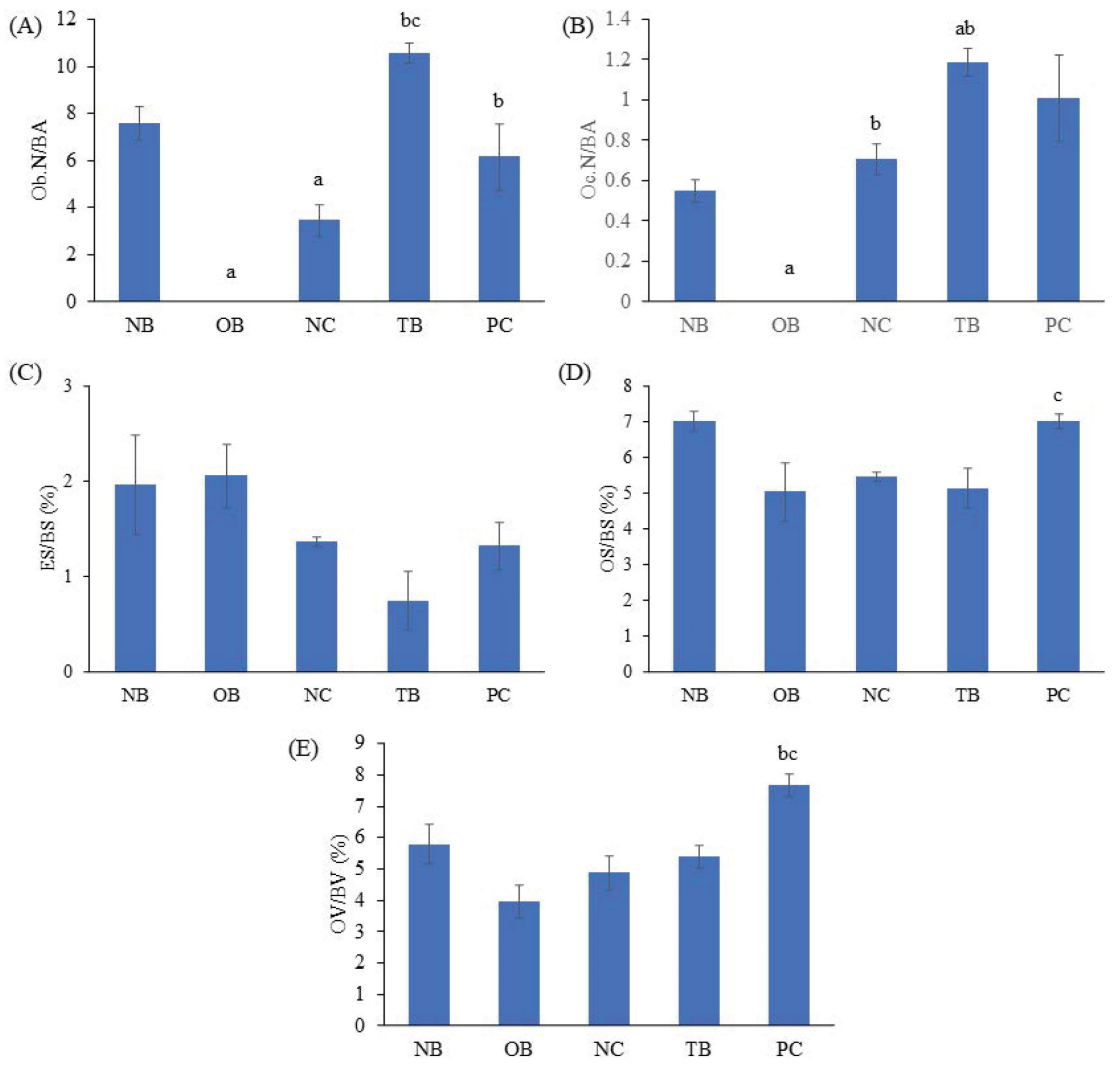
Effects of palm carotene mixture (12.5 µg/mL) on (A) Ob.N/BA, (B) Oc.N/BV, (C) ES/BS, (D) OS/BS, and (E) OV/BV of bone scaffold co-cultured with osteoblasts and osteoclasts for 21 days after H&E staining. Data are expressed as mean ± SEM. 'a' indicates significant difference with NB; 'b' indicates significant difference with OB; 'c' indicates significant difference with NC; 'd' indicates significant difference with TB. Abbreviations: NB, native bone scaffold; OB, osteoporosis bone scaffold; NC, negative control bone scaffold; TB: 12.5 μg/mL palm carotene mixture-treated bone scaffold; PC, 10 nM alendronate-treated bone scaffold.

**Figure 12:**
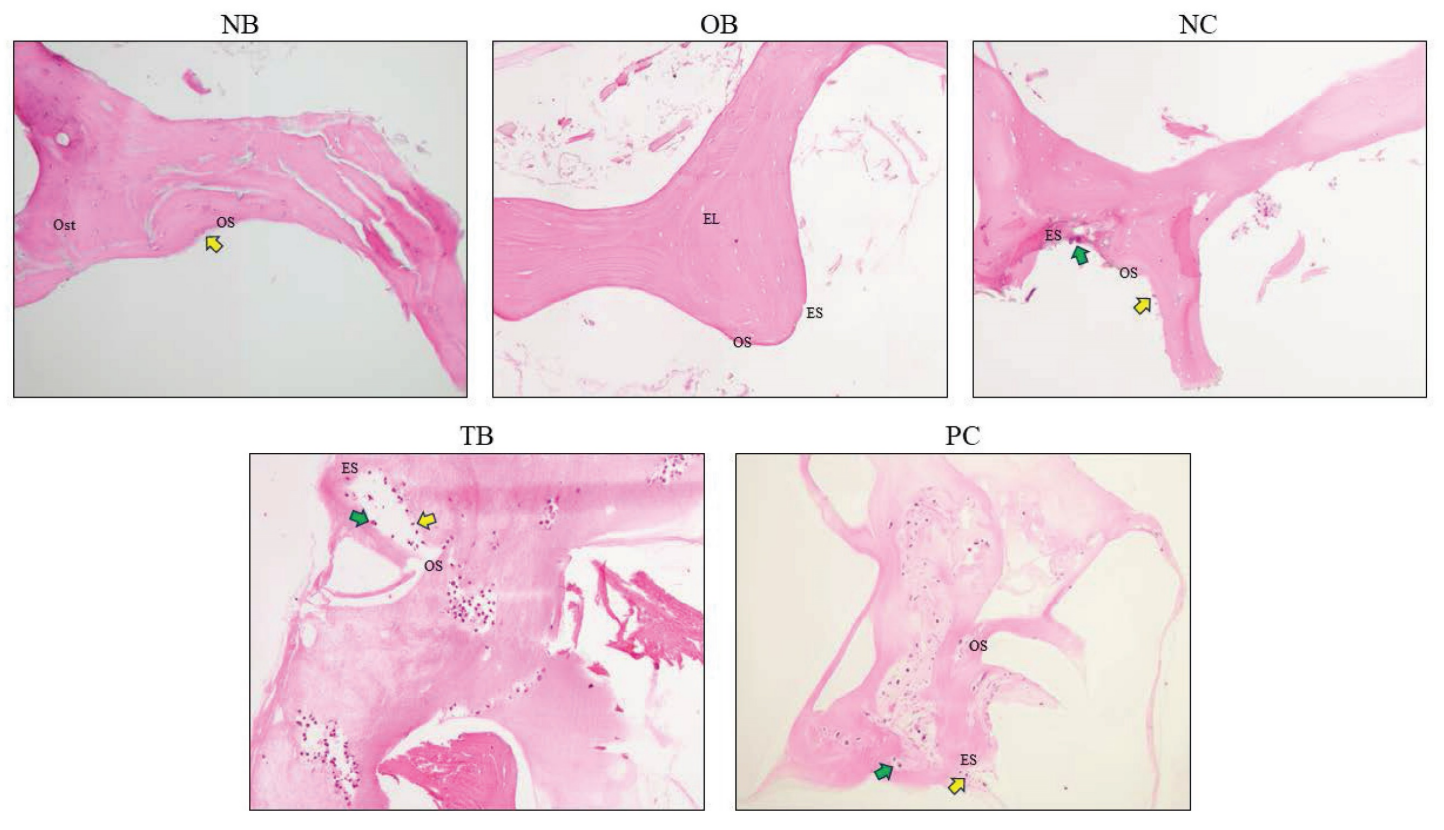
Representative images of bone scaffold after H&E staining before decellularisation and demineralisation and after treatment of 21 days. The yellow arrows indicate osteoblasts and green arrows indicate osteoclasts. Abbreviations: NB, native bone scaffold; OB, osteoporosis bone scaffold; NC, negative control bone scaffold; TB: 12.5 μg/mL palm carotene mixture-treated bone scaffold; PC, 10 nM alendronate-treated bone scaffold; Ost, osteocyte; OS, osteoid surface; ES, eroded surface; EL, empty lacunae.

The effects of palm carotene mixture (12.5 µg/mL) on Ob.N/BA, Oc.N/BA, ES/BS, OS/BS, and OV/BV of bone scaffolds seeded with osteoblasts and osteoclasts after H&E staining were evaluated. The Ob.N/BA and Oc.N/BA were reduced in the osteoporotic bone scaffolds (OB) as compared to the native bone scaffolds (NB) (p<0.05). The negative control bone scaffolds (NC) showed significant higher Oc.N/BA in comparison to the osteoporotic bone scaffolds (OB) (p<0.05). Bone scaffolds treated with palm carotene mixture (TB) indicated higher Ob.N/BA in relative to the negative control bone scaffolds (NC) (p<0.05). Bone scaffolds treated with alendronate (PC) showed significant increase in OS/BS and OV/BV as compared to the negative control bone scaffolds (NC) (p<0.05) (Fig. 11). The representative of H&E staining images observed under microscope for all groups were presented (Fig. 12).

## Discussion

In this study, the cytotoxic effects of palm carotene mixture were determined on hFOB 1.19 and THP-1 cells. Osteoblast cells were viable in the presence of palm carotene mixture at concentrations ranged from 3.13 - 50 μg/mL. Palm carotene mixture at lower concentration (3.13 - 12.5 μg/mL) decreased but higher concentration (25 - 50 μg/mL) increased the viability of THP-1 cells. A two-dimensional osteoblast/osteoclast co-culture system was used to determine the effects of palm carotene mixture on hFOB 1.19 proliferation and osteoclast maturation differentiated from THP-1 cells. The results indicated that co-culture cells treated with 12.5 µg/mL palm carotene mixture hastened the proliferation of osteoblasts (evidenced by earlier peak of ALP-positive area) but inhibited maturation of osteoclasts (evidenced by increased TRAP-positive multinucleated cells).

The hFOB 1.19 cells are adherent cells transfected from foetal limb tissue of spontaneous miscarriage, having the ability to differentiate into mature osteoblasts by expressing the typical osteoblast phenotype. Our study demonstrated that treatment of palm carotene mixture dose-dependently increased the viability of hFOB 1.19 cells from day 1 to day 6. This observation was in line with a previous study demonstrated that the pre-osteoblasts were viable in the presence of β-carotene 5. On the other hand, THP-1 cells are suspension cells isolated from peripheral blood obtained from male acute monocytic leukaemia patient, with the capability to differentiate into osteoclasts upon stimulation with RANKL 25. The data from current study showed that the cell viability increased from day 1 to day 3 and decreased from day 3 to day 6 in vehicle-treated THP-1 cells, possibly attributed to the short lifespan of monocytes approximately between 4 to 7 days 26. Higher concentrations of palm carotene mixture increased while lower concentrations of palm carotene mixture decreased the viability of THP-1 monocytes were also observed in this study. In accordance to current findings, a previous study using bone marrow-derived monocytes/macrophages stimulated by RANKL found that β-carotene at the concentration of 0.4 to 0.6 μM reduced cell viability after five days 7. Hence, β-carotene inhibited the viability of monocytes/macrophages, thus potentially lead to lower number of active osteoclasts. Based on the results obtained in this study, palm carotene mixture at the concentrations of 3.13, 6.25, and 12.5 µg/mL have been selected to assess their effects on osteoblast differentiation and osteoclast maturation using a two-dimensional osteoblast/osteoclast co-culture system.

Bone is an active and dynamic organ undergoing physiological bone remodelling, including the process of bone resorption by osteoclasts balanced by bone formation by osteoblasts 27. Osteoblast/osteoclast co-culture system mimics the bone's physiological condition in human allowing the interaction between osteoblasts and osteoclasts in the regulation of bone formation and resorption processes 28. Bone ALP is highly expressed in osteoblasts as an early gene marker. The level of ALP peaks around day 7 to 14, indicating the active phase of osteoblast differentiation and new bone matrix formation 29-31. Histomorphometrically, the osteoblasts with ALP-positive sites appear blue. The ALP-positive area was increased on day 14 when the co-culture cells were treated with 12.5 µg/mL palm carotene mixture as compared to the control in our current study. Similarly, study by Nishide *et al.* demonstrated higher ALP activity in MC3T3-E1 pre-osteoblastic cells treated with a combination of isoflavones and β-carotene 6. In addition, an earlier peak for ALP activity was observed on day 14 after treatment with 12.5 µg/mL palm carotene mixture, which was not seen in other treatment groups. This finding reiterated that 12.5 µg/mL palm carotene mixture hastened osteoblast differentiation. ALP facilitates bone mineralisation by hydrolysing extracellular pyrophosphate to increase local inorganic phosphates 32. Thus, earlier peak of ALP expression enhanced earlier bone mineralisation.

In osteoblast/osteoclast co-culture system, the secretion of M-CSF and RANKL by osteoblasts allows the fusion of mononuclear progenitors (THP-1 monocytes) forming multinucleated cells and eventually activating the osteoclasts. TRAP is highly expressed in osteoclasts to digest minerals and organic components of bone matrix along with other proteinase (such as cathepsin K, matrix metalloproteinase, and gelatinase) forming the resorption pits. Therefore, TRAP is commonly used as a specific histochemical marker for osteoclasts and bone resorption activity 33. Histomorphometrically, the mature osteoclasts appear as purplish red giant multinucleated cells after TRAP staining. This current study demonstrated that TRAP-positive cells were evident on day 7 in the co-culture system. In another study, multinuclear cells were also seen after THP-1 cells stimulated for 6 days with RANKL 25. The results of present study also pointed out that the number of multinucleated giant cells was not affected at lower concentration of palm carotene mixture throughout the study period, but inhibition of active osteoclast formation was noted at higher concentration (12.5 µg/mL) on day 21. A similar trend was found using bone marrow-derived monocytes/macrophages exposed to RANKL, whereby the reduction of TRAP-positive multinucleated cells was seen after 72 hours of incubation with β-carotene in higher concentration (0.1 - 0.6 μM) but not in lower concentration (0.05 μM) 7. In brief, palm carotene mixture at 12.5 µg/mL hastened osteoblast proliferation and suppressed osteoclast maturation in the two-dimensional co-culture system. Thus, this concentration has been selected as the treatment in subsequent three-dimensional co-culture system involving the seeding of osteoblasts and osteoclasts on bovine bone scaffolds.

Cells grown in three-dimensional culture are more physiologically relevant for cell growth, proliferation, differentiation, morphology, and cellular functionality because the culture environment that allows the interaction with surrounding extracellular framework. On the other hand, the traditional two-dimensional cell culture allows cells to grow in flat monolayer on culture plate 34. In skeletal microenvironment, the crosstalk of bone-forming osteoblasts and bone-resorbing osteoclasts is a fundamental requirement for balanced bone remodelling 35. Trabecular bone is a porous interconnected network facilitating cell infiltration, migration, proliferation, nutrient and oxygen diffusion 36. In this study, an *in vitro* three-dimensional co-culture system was established by seeding hFOB 1.19 cells and THP-1 monocytes on decellularised and demineralised bovine bone scaffolds to mimic human endogenous skeletal microenvironment. This system can be used to unravel the effects of palm carotene mixture on bone. The assessments of BMC, BMD, trabecular microstructure, biomechanical strength, and histomorphometry of bone scaffolds after bone cells seeding and palm carotene treatment were performed. A similar three-dimensional co-culture system has been previously developed by Jolly *et al.* via the seeding of hFOB 1.19 cells and human peripheral blood mononuclear cells on bovine bone scaffold for 21 days. Their findings indicated that bone cells are structurally, functionally, and mechanically comparable with those in the natural bone 20.

The BMC and BMD of the bone scaffolds were determined after treatment of palm carotene mixture for 21 days. BMC represents total mineral content in bone while BMD refers to the total mineral content at specific areas of the bone 37. Our study showed that the BMC and BMD of bone scaffolds showed no significant difference before and experiment in all groups. These observations might be due to low resolution of DXA machine to trace changes in the small-sized bone scaffold samples. Micro-CT analysis utilises high resolution images to observe three-dimensional bone structure and evaluates the geometry of bones. Common parameters used to identify trabecular bone microstructure includes BV/TV, Tb.N, Tb.Th, Tb.Sp, and porosity (Bouxsein *et al.* 2010). The results of current study showed no significant change when bone scaffolds were treated with palm carotene mixture. Previous *in vivo* study indicated no significant results in all bone microstructure parameters in diabetic rats induced by streptozotocin when treated with spirulina containing β-carotene 38. The micro-CT data also spectated significant increase in Tb.Th when treated with alendronate compared to the negative control. Our results were in line with a study carried out by Tokmak Özşahin *et al.* whereby significant increase in Tb.Th was also seen in ovariectomised rats treated with alendronate 39.

Bone quality describes the mineral composition and bone microstructure that contribute to bone strength 40. Bone biomechanical strength can be classified into extrinsic and intrinsic characteristics to describe the general integrity of bone. Extrinsic characteristics such as load, stiffness, and displacement represent external bone strength which is influenced by density, microarchitecture and characteristics of the bone. Intrinsic characteristics such as stress, strain and Young's modulus resembles internal bone strength which is influenced by bone tissues. Load and stress measure the maximum force needed to fracture a bone. Stiffness and Young's modulus represent bone resistance towards deformation. Displacement and strain are the measure for bone ductility, which is the maximum deformation a whole bone can undergo before fracture occurs 41. Decellularisation and demineralisation processes increased displacement and strain in current study, possibly due to the removal of calcium content in bone through demineralisation process. The seeding of bone cells into the decellularised and demineralised scaffolds showed lower displacement and strain. This might be because of the presence of cells which provides bone formation and bone resorption events to restore the calcium content in bone scaffolds. This present study also showed no significant outcomes in bone biomechanical strength parameters when treated with palm carotene mixture. Likewise, an *in vivo* study carried out by Matsumoto *et al.* reported no significant difference for biomechanical strength parameters between the hindlimb unloading mice fed with normal diet and β-carotene-supplemented diet 4.

Bone histomorphometry analysis were carried out on the three-dimensional bone scaffolds infused with bone cells. Von Kossa staining for undecalcified bone detects mineral deposits and illustrates mineralisation in bone. The two-step reaction in von Kossa staining involves the reaction of silver cations (in silver nitrate solution) with phosphates (the components of calcium deposits in bone) resulting in yellowish brown colouration, followed by reduction of bound silver to metallic black silver after exposure to light 42. In this study, palm carotene mixture increased BV/TV of bone scaffolds seeded with osteoblasts and osteoclasts. Previous study investigating the effects of carotene on structural bone histomorphometry is limited. Although micro-CT scanning and von Kossa staining assess bone microstructure, the different results yielded from both techniques might be due to difference in region of interest selected for analyses. Micro-CT scanning involves contouring on 200 slices of bone images to be analysed whereas von Kossa staining emphasises on several slices of longitudinal sections to be observed under the microscope. The H&E staining provides static bone histomorphometry parameters to quantify osteoblasts, osteoclasts, unmineralised bone (osteoid), and degree of bone resorption at trabecular region 43. An increase in Ob.N/BA was observed in this study when bone scaffolds were treated with palm carotene. Limited research was done to access the effects of carotene on static bone histomorphometry. After treated with alendronate, Ob.N/BA, OS/BS, and OV/BV of bone scaffolds were increased in this study. These results were supported by previous findings by Tokmak Özşahin *et al.* indicating the improvement in bone histomorphometric parameters such as mineralised bone and osteoid volume in ovariectomised rats treated with alendronate 39.

Palm carotene mixture was used in this study because it exists as complex mixtures of various isomers naturally. It represents a more cost-effective option as compared to isolated individual carotene isomers. In adults, the recommended daily allowance (RDA) of vitamin A is expressed in retinol activity equivalents (RAE): 900 μg for men, 700 μg for women, 770 μg for pregnant individuals, and 1300 μg for those who are lactating. Each μg of RAE corresponds to 1 μg of retinol, 12 μg of dietary beta-carotene, or 24 μg of dietary α-carotene or beta-cryptoxanthin 44. The ratio for α-carotene, β-carotene, and other carotenoids in the mixture is 33:66:1. Thus, the maximum recommended dose for palm carotene mixture is approximately 14 mg daily for men and 11 mg daily for women. The toxicity of β-carotene is minimal. Animal studies indicated that β-carotene is not mutagenic, embryotoxic, tumourigenic, carcinogenic, teratogenic, and does not cause hypervitaminosis A 45. Clinical trials have confirmed the safety of β-carotene supplementation in doses ranging from 15 to 50 mg per day 46. However, prolonged intake of β-carotene supplements exceeding 30 mg per day may result in hypercarotenaemia (a yellowing of the skin), which typically resolves after discontinuing the treatment 45.

Alendronate is the first line therapy for osteoporosis. It serves as an ideal positive control for in vitro or in vivo experimental designs in osteoporosis-related studies, allowing the researchers compare experimental treatments to a drug with known efficacy in humans. Alendronate (0.1 to 10 nM) reduced the expression of TRAP and CTSK in RANKL-induced osteoclastogenesis 47. In addition, alendronate has a clear mechanism of action in suppressing osteoclast-mediated bone resorption by decreasing the RANKL/OPG ratio 48. Alendronate is also inexpensive and readily available for research purposes. Hence, it provides a reliable benchmark to assess the bone-protecting effects of novel interventions.

Several limitations were acknowledged in this study. Firstly, Verhoeff-van Gieson and Alizarin Red staining were not done in a two-dimensional co-culture system to detect the presence of collagen and mineral content in bone, respectively. Secondly, TRAP staining was not conducted in the three-dimensional co-culture system to identify mature osteoclasts and confirm the identification of osteoclasts based on cellular morphology in H&E staining images. Thirdly, the measurement of bone markers was not performed thus the mechanism of action underlying the action of palm carotene mixture was not elucidated. The strength of current study includes the use of osteoblast/osteoclast co-culture that resembles the dynamic physiological process of bone remodelling executed through coupled activities of osteoclasts and osteoblasts in human. No external stimulation or inducer (such as M-CSF and RANKL) are required as they can be produced naturally by mature osteoblasts 19. In addition, the decellularisation process eliminates cellular components from native bovine bone to offer an improvement over the previously developed demineralised bone scaffolds 20 and serve as a promising structural extracellular matrix template with biomimetic microenvironment 49, 50. Hence, the seeding of osteoblasts and osteoclasts on decellularised and demineralised bone scaffold bring a step closer in mimicking the human skeletal microenvironment. This study highlighted the potential of palm carotene in inducing earlier osteoblast proliferation and inhibiting osteoclast activation in monolayer co-culture. Although the observable changes in three-dimensional co-culture system were limited to increased bone volume and osteoblast number, it is postulated that longer treatment duration may be needed to detect significant changes in other parameters. It takes at least 21 days for osteoblasts to differentiate and mineralise 51, thus limited bone changes were noted in a cycle of bone remodelling. Besides, osteoblasts and osteoclasts may respond differently in two- and three-dimensional culture environment. Cell-cell interactions are simpler in two-dimensional monolayer culture whereby bone markers diffuse more easily to support cell proliferation and activation. In three-dimensional culture environment, the cell-cell and cell-matrix interactions are more physiologically relevant but may dampen the response to treatment. Further studies are warranted to optimise the time required to detect bone changes after treatment of palm carotene. Harvesting of bone scaffolds at different time points to measure the bone parameters after treatment can be performed.

## Conclusion

In a two-dimensional co-culture system, palm carotene mixture hastens osteoblast proliferation while suppressing osteoclast maturation. In a three-dimensional co-culture system, the bone-protecting actions of palm carotene mixture were evidenced by higher bone volume and osteoblast number. Findings from current study suggest that palm carotene potentially enhances osteoblastic bone formation and inhibits osteoclastic bone resorption, which await further validation from *in vivo* studies and human trials.

## Abbreviations

ALP: alkaline phosphatase
ANOVA: analysis of variance
BMC: bone mineral content
BMD: bone mineral density
BV/TV: bone volume/tissue volume
CO_2_: carbon dioxide
DMEM/F-12: Dulbecco's Modified Eagle Medium/Nutrient Mixture F-12
DXA: dual energy X-ray absorptiometry
EDTA: ethylenediaminetetraacetic acid
ES/BS: eroded surface/bone surface
hFOB 1.19: human foetal osteoblasts
H&E: haematoxylin and eosin
M-CSF: macrophage colony-stimulating factor
micro-CT: micro-computed tomography
MTT: 3-(4,5-dimethylthiazol-2-yl)-2,5-diphenyl-2H-tetrazolium bromide
NB: native bone scaffold
NC: negative control bone scaffold
OB: osteoporotic bone scaffold
Ob.N/BA: osteoblast number/bone area
Oc.N/BA: osteoclast number/bone area
OS/BS: osteoid surface/bone surface
OV/BV: osteoid volume/bone volume
PC: 10 nM alendronate-treated bone scaffold
RANKL: receptor activator of nuclear-kappa B ligand
REC: Research Ethics Committee
SEM: standard error of mean
SPSS: Statistical Package for the Social Sciences
TB: 12.5 μg/mL palm carotene mixture-treated bone scaffold
Tb.N: trabecular number
Tb.Sp: trabecular separation
Tb.Th: trabecular thickness
THP-1: human monocytic cell line
TRAP: tartrate resistant acid phosphatase
